# An *in vitro* diagnostic certified point of care single nucleotide test for *IL28B* polymorphisms

**DOI:** 10.1371/journal.pone.0183084

**Published:** 2017-09-06

**Authors:** Darragh Duffy, Estelle Mottez, Shaun Ainsworth, Tan-Phuc Buivan, Aurelie Baudin, Muriel Vray, Ben Reed, Arnaud Fontanet, Alexandra Rohel, Ventzislava Petrov-Sanchez, Laurent Abel, Ioannis Theodorou, Gino Miele, Stanislas Pol, Matthew L. Albert

**Affiliations:** 1 Immunobiology of Dendritic Cells, Institut Pasteur, Paris, France; 2 Inserm U1223, Institut Pasteur, Paris, France; 3 Centre for Translational Research, Institut Pasteur, Paris, France; 4 Inserm UMS20, Institut Pasteur Paris, France; 5 Genedrive plc, Manchester, United Kingdom; 6 Université Paris Descartes et Département d'hépatologie, Groupe Hospitalier Cochin Hôtel- Dieu, Paris, France; 7 Emerging Disease Epidemiology Unit, Institut Pasteur, Paris, France; 8 PARCI Unit, Conservatoire National des Arts et Métiers, Paris, France; 9 Unit of epidemiology of infectious diseases, Institut Pasteur, Dakar, Senegal; 10 ANRS (France REcherche Nord&Sud Sida-hiv Hépatites), Paris, France; 11 Laboratory of Human Genetics of Infectious Diseases, Necker Branch, Inserm U1163, Paris, France; 12 Paris Descartes University, Imagine Institute, Paris, France; 13 St. Giles Laboratory of Human Genetics of Infectious Diseases, Rockefeller Branch, Rockefeller University, New York, New York, United States of America; 14 Department of Immunology, AP-HP, La Pitie Salpetriere, Paris, France; Centre de recherche du CHUM, CANADA

## Abstract

Numerous genetic polymorphisms have been identified as associated with disease or treatment outcome, but the routine implementation of genotyping into actionable medical care remains limited. Point-of-care (PoC) technologies enable rapid and real-time treatment decisions, with great potential for extending molecular diagnostic approaches to settings with limited medical infrastructure (e.g., CLIA certified diagnostic laboratories). With respect to resource-limited settings, there is a need for simple devices to implement biomarker guided treatment strategies. One relevant example is chronic hepatitis C infection, for which several treatment options are now approved. Single nucleotide polymorphisms (SNPs) in the *IL-28B* / *IFNL3* locus have been well described to predict both spontaneous clearance and response to interferon based therapies. We utilized the Genedrive^®^ platform to develop an assay for the SNP rs12979860 variants (CC, CT and TT). The assay utilizes a hybrid thermal engine, permitting rapid heating and cooling, enabling an amplification based assay with genetic variants reported using endpoint differential melting cure analysis in less than 60 minutes. We validated this assay using non-invasive buccal swab sampling in a prospective study of 246 chronic HCV patients, achieving 100% sensitivity and 100% specificity (95% exact CI: 98.8–100%)) in 50 minutes as compared to conventional lab based PCR testing. Our results provide proof of concept that precision medicine is feasible in resource-limited settings, offering the first CE-IVD (in vitro diagnostics) validated PoC SNP test. We propose that *IL-28B* genotyping may be useful for directing patients towards lower cost therapies, and rationing use of costly direct antivirals for use in those individuals showing genetic risk.

## Introduction

The sequencing of the human genome followed by the subsequent revolution in sequencing technologies heralded an ambition for precision medicine [1]. Actual translation into clinical applications, however, has remained slow as illustrated by the low number of FDA approved pharmacogenomic biomarkers [2], as compared to protein based biomarker assays [3]. One challenge has been the implementation of molecular based diagnostics into routine clinical use, with even greater hurdles for resource-limited settings. Point-of-care (PoC) technologies enable rapid and real-time treatment decisions, with potential for extending molecular diagnostic approaches to settings with limited medical infrastructure such as a CLIA (Clinical Laboratory Improvement Amendments) certified diagnostic laboratories or decentralized laboratory settings [4].

Chronic infection with the hepatitis C virus (HCV) can result in liver failure, cirrhosis or liver cancer with an estimated 150 million people infected worldwide [5]. For patients with HCV, the major clinical question concerns the mode of treatment, with the recent introduction of several direct acting antivirals (DAAs)[6]. The high cost of these treatments, however, has required many countries to restrict treatment to those patients with advanced disease [7]. The use of diagnostic predictive tests therefore remains relevant for the selection of a suitable treatment plan for each patient, with particular relevance for persons in resource-limited settings. Genetic factors have been identified as predictors of response to treatment, in particular polymorphisms of the *IL28B* gene [8],[9]. The most well characterized locus is the rs12979860 SNP, with 3 possible genotypes: CC, CT, and TT. The CC genotype has been demonstrated to predict better virological response rates to interferon based therapies [10], with its utility for DAAs remaining to be defined. Genotyping is currently performed using classical PCR amplification applied to DNA extracted from blood or plasma, with a time to result of 2–3 weeks, thus limiting its clinical implementation.

Point-of-care technologies are having increasing importance in medical diagnostics, with applications in settings with limited medical infrastructure [11]. The Cepheid PCR Xpert ^®^ MTB/RIF test[12] is one example of successful deployment of molecular based technology for pathogen testing [13],[14]. More recently, Genedrive plc has developed a CE-marked molecular diagnostic platform that is capable of performing ultra-fast PCR cycling and rapid actionable results. We developed a Genedrive^®^ SNP test for *IL28B* using buccal swabs with no requirement for nucleic acid isolation. Herein we describe a prospective clinical study, testing HCV patients that compared a rapid non-invasive buccal swab test to the standard blood DNA based test. We achieved 100% identity for the results from the two assays, validating this novel technology and demonstrating its readiness for use in PoC testing. Implementation of this methodology for SNP testing will enable efficient and decentralized patient management solutions.

## Materials and methods

### Patient cohort

This study was conducted in accordance with International Council on Harmonization Good Clinical Practice guidelines and was approved by an independent ethics committee (CPP Ile-de-France VI) who classified the study as non-interventional. The ANRS (France REcherche Nord&Sud Sida-hiv Hépatites) as study sponsor was responsible for the regulatory and administrative aspects. All patients provided written informed consent before enrollment on the basis of the ethical principles outlined in the Declaration of Helsinki. Patients were considered eligible if they were aged 18 years or older, diagnosed with hepatitis C (all viral genotypes), and either already treated, or currently undergoing antiviral therapy, or recommended to start antiviral therapy against hepatitis C virus. In addition, patients had already consented to *IL-28B* genotyping in the course of their usual follow-up by the conventional PCR standard method. All patients were recruited at a single site (Hepatology Department, Cochin Hospital, Paris, France) and the trial was registered on clinical trials.gov (NCT02335320). 246 patients were recruited over a period of 6 months and patient characteristics are detailed in Table 1.

**Table 1:** Patient demographics and baseline characteristics.

Parameters	N = 246
**Male**, n(%)	149 (61%)
**Age**	
Mean +/- SD	57 +/- 11
Min, Max] (years)	[21, 87]
**Fibrosis Status, n (%)**	
**F1**	81 (33%)
**F2**	37 (15%)
**F3**	69 (28%)
**F4**	59 (24%)
**Treatment, n (%)**	
Naïve	108 (44%)
Experienced	117 (48%)
Ongoing	16 (7%)
Cured	5 (2%)
**Viral genotype, n (%)**	
**G1**	160 (65%)
**G2**	14 (6%)
**G3**	28 (11%)
**G4**	40 (16%)
**G5**	3 (1%)
**G6**	1 (0%)
**IL-28B Genotype**	
CC, n (%)	76 (31%)
CT, n (%)	134 (54%)
TT, n (%)	36 (15%)

### Sample size estimation

In order to show a sensitivity and a specificity of at least 85% and given expected sensitivities and specificities of the assay around 95% and a power of 80%, the number of cases (CC) required was 76 and the number of controls (Non CC) was also 76. Assuming that the proportion of cases among suspects will be around 35%, the number of controls will be approximately 174 and therefore to be sure of sufficient power for such analysis 250 patients were include in the study[15].

### Genedrive platform

The Genedrive^®^ platform is a single wavelength rapid PCR thermocycler that performs real time nucleic acid amplification and end-point melt analysis detection[16]. It is a small (600g, 18x12x10cm), stand-alone field-deployable diagnostic instrument for the rapid, multiplex detection of infectious agents in a range of sample matrices in <90mins per sample. Using real-time fluorogenic polymerase chain reaction (PCR) with end-point melt analysis, it is capable of analyzing 5–6 samples per day without reliance on external IT or specialist laboratory equipment. With a simple graphical user interface and single button operation, Genedrive fully automates test cycling, data analysis and diagnostic results interpretation from the moment the test cartridge is introduced. Genedrive uses a single wavelength optical system (400-470nm LEDs, 535nM photodiodes) used to read a PCR test cartridge, comprised of 3 reaction wells. Each well can accommodate up to 45ul reaction volume and discriminate up to 4x RNA/DNA targets (or 3 + internal controls) by melting temperature of specific fluorescein-labelled Hybeacon-type molecular probes. As such, up to 12 targets can be detected in parallel per cartridge, to allow the ability to screen multiple targets per organism for genetic typing or drug susceptibility. Connectivity with external devices is provided via a micro-USB and Bluetooth although the system can be operated in decentralized settings without any reliance on peripheral equipment. Additional technical specifications are available here https://www.genedrive.com/genedrive-system/documentation.php. The *IL28B* SNP assay described here is an asymmetric PCR assay, with endpoint melt temperature analysis providing allelic discrimination via fluorescent hybridization probes.

### Genedrive^®^ assay testing

A Clinical Research Assistant was assigned to perform the Genedrive^®^ IL-28B genotyping assays. Four units were set up which enabled twelve patients to be tested daily, with each machine testing a single patient (triplicate samples) in a 1 hour period. Following consent, a buccal swab was performed by the nurse or the patient themselves and analysed promptly with the Genedrive^®^ unit according to the operating instructions. Briefly, 20μl of the buccal swab sample was dispensed into the neck of each tube of the Genedrive^®^ cartridge using a micropipette. The cartridge was then placed into the Genedrive^®^ unit and the genotype analysis initiated. Each test was performed in triplicate within one sample cartridge. To achieve a confirmed result at least 2 of 3 tests must return concordant results. If conflicting results were achieved from the triplicate tests the unit performed a repeat analysis, after which if the triplicate results remained in disagreement a “fail” was returned. All failed tests were noted and reported.

### TaqMan PCR SNP genotyping

Whole blood was collected in heparin tubes, from which DNA was extracted (Qiagen DNAeasy blood and tissue kit) and analyzed using TaqMan SNP genotyping assays (Applied Biosystems Inc., Foster City, CA) for the rs12979860 SNP.

### Statistical analysis

Genedrive test result data were automatically printed, and attached to a daily test results log, and entered daily into a study-specific electronic case report form (e-CRF). An independent study investigator entered the TaqMan SNP genotyping results in order to maintain blinding of results prior to comparison. The database was checked for completeness before performing the analysis. To achieve the main objective, the results of the assay (CC / non CC) was based on a 90% rectangular confidence region using one-sided exact confident limits. The Cohen’s kappa coefficient was used to evaluate the agreement between the index test and the TaqMan SNP genotyping results expressed as CC, CT and TT. Full data set is available at 10.6084/m9.figshare.5328721.

## Results

### Establishment of a PoC SNP test for *IL28B* polymorphisms

**Fig 1:**
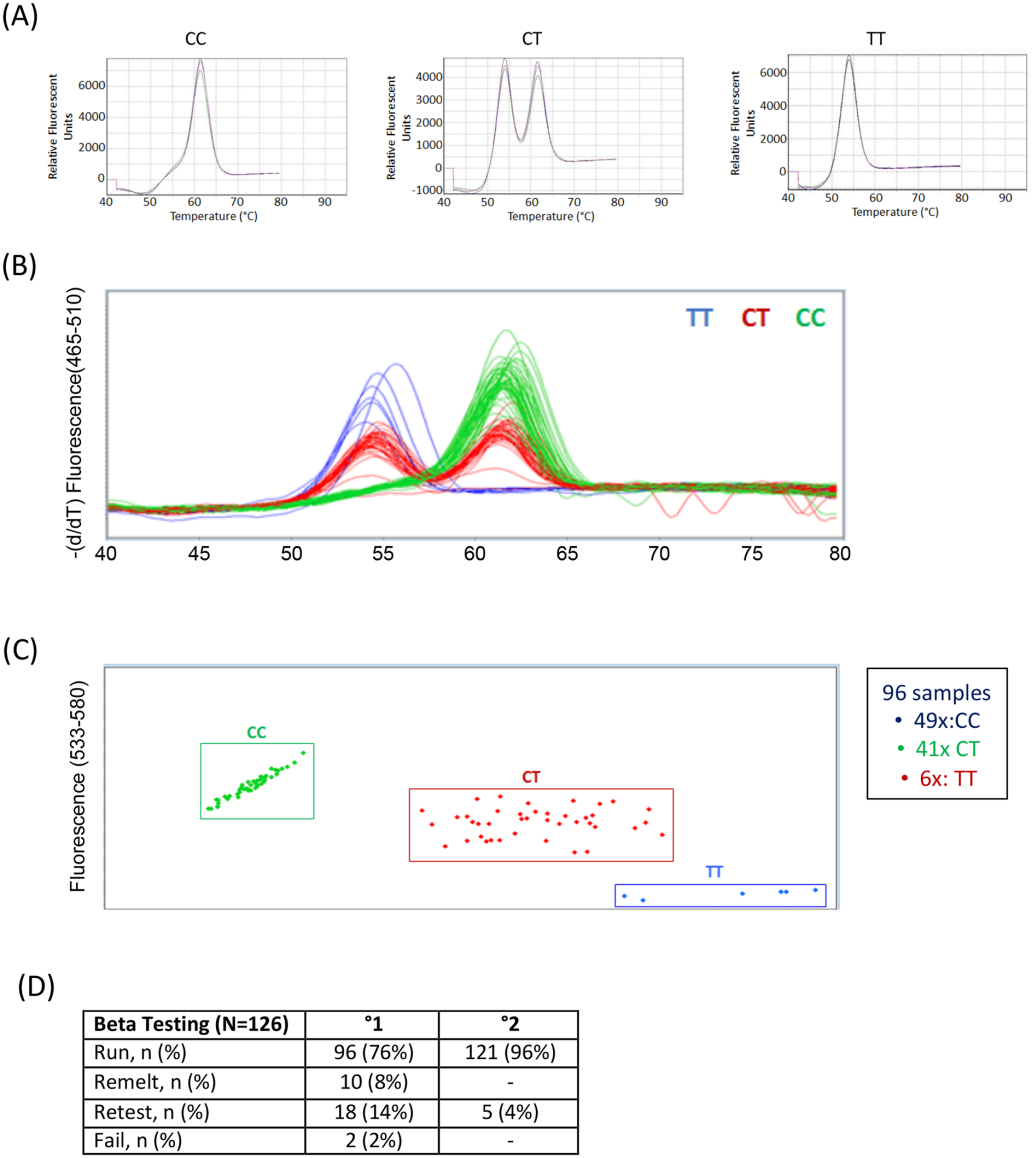
Comparison of Genedrive^®^ and Taqman assays. (A) Melt Curve Analysis for detection of SNP and template control. (B) Results of Genedrive assay on initial test samples. (C) Results of Taqman assay on initial test samples. (D) Summary of initial Beta test results in run, remelts, and retests prior to and after assay optimization.

Genedrive^®^ utilizes asymmetric PCR and a thermal cycling engine for rapid heating and cooling. It takes advantage of fluorescent probe based endpoint differential melt curve analysis to detect the two genotypic alleles. Primers were designed to identify the rs12979860 locus and a fluorescent probe to discriminate the rs12979860 SNP of *IL28B*. Upon amplification of the respective alleles they were identified based on different melting peaks: C allele having a median melting temperature of 62°C, and T with a median melting temperature of 53°C, and the heterozygous CT alleles presenting peaks at both temperatures (Fig 1A). Assay verification studies were performed following optimization against buccal swab matrix, which showed the test to have an analytical limit of detection (LOD) of 100 copies of genomic DNA, and current real time stability studies show a shelf life of 18 months of the lyophilized product. The assay was shown to tolerate all common interfering substances that were tested, especially after a mouth rinse, when using fresh buccal samples. Analytical accuracy was observed to be 100% following the analysis of 96 random samples using the Genedrive IL28B test, with the resulting genotypes shown in Fig 1B, and the confirmatory TaqMan PCR results shown in Fig 1C. For development purposes and initial testing of the Genedrive assay, we performed a clinical study, collecting 126 patient buccal samples from HCV patients. Testing resulted in accurate results from 96 samples (76%), with 10 (8%) giving an accurate result following a remelt, 18 (14%) requiring a complete retest, and 2 samples failing to give a result (Fig 1D). To rectify this, the lysis buffer and cycling parameters were adjusted to achieve a more intense melting curve signal. Following these modifications and using these optimized conditions, all 121 (96%) samples returned a result from a new initial run, with sample re-test results been reduced to 4% of total runs (Fig 1D).

### Clinical study validation of the PoC SNP test for *IL28B*

**Fig 2:**
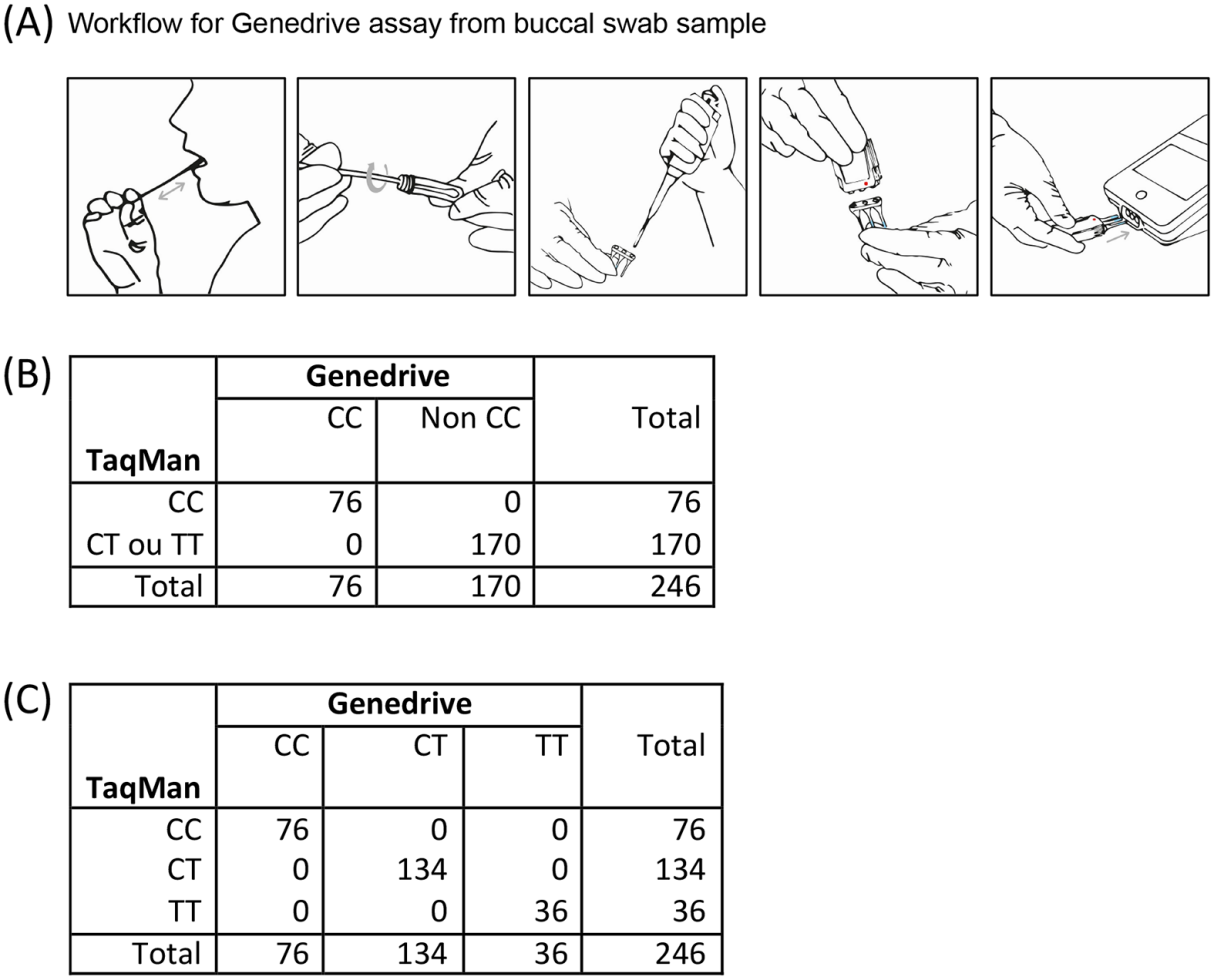
Clinical study validation of 246 HCV patients. (A) Workflow and Layout of Assay. (B) Contingency table for sensitivity and specificity to detect CC genotype. (C) Contingency table for sensitivity and specificity to discriminate CC, CT, and TT genotypes.

Upon establishment of the PoC SNP test we set out to validate its accuracy in a prospective clinical study. Patients chronically infected with HCV were recruited and buccal swabs were used for real-time testing in the outpatient unit of the Hepatology Department of Hopital Cochin, Paris. Patients were recruited as they attended the clinic for routine monitoring and upon signing consent forms were asked to prepare their own buccal swab as illustrated in Fig 2A. Following a mouth rinse with 30ml water the fat part of the swab was rubbed across the donors inner cheek 10 times in a forward/backward motion. This was repeated using the reverse side of the swab on the opposite cheek (Fig 2A). The swab tip was submerged in 1.8ml lysis buffer provided with the assay kit, mixing for 30 seconds being performed by the clinical research assistant, and then discarded (Fig 2A). 20μl of the buccal sample mix was then added to the neck of each tube of the Genedrive cartridge, the lid manually locked shut and the cartridge was inserted into the Genedrive device for analysis (Fig 2A). Results (total analysis time) were obtained in less than 60 minutes, printed out and attached to the case report form. In parallel, blood was collected from the patient, transported to a clinical testing lab (La Pitié Salpêtrière) where DNA was extracted for the reference TaqMan genotyping analysis. Results from the centralized laboratory were typically returned within 1–2 weeks, but kept blinded until patient recruitment was completed.

### Statistical comparison of Genedrive and Taqman results

The first objective of the study was to compare the ability of Genedrive to correctly identify the CC genotype, as distinct from the CT and TT genotypes. Out of the 246 patients, 76 donors showed only one peak, which corresponded to the C alleles and were thus classified as being CC genotype. 170 donors showed a peak corresponding to the T allele and were thus classified as being non-CC genotype. There results were independently confirmed using conventional assays, giving 100% sensitivity and 100% specificity with 95% confidence intervals (CI) of 98.8–100% (Fig 2B). The second objective of the study was to discriminate the 3 individual genotype, and both techniques identified 76 CC genotypes, 134 CT genotypes, and 36 TT genotypes, in matched donors giving a Cohen’s kappa coefficient of 1 (Fig 2C). Regarding the technical aspects of the assay, for the 246 patients tested in the clinical study, Genedrive returned a result from the first run for 97% of the samples, with 3 samples passing after a further remelt of the primary sample (1%), and 4 samples requiring a retest of the provided buccal swab material (2%) (Table 2).

**Table 2:** Summary of clinical test results in runs, remelts, and retests.

Test results	N = 246
Run, n (%)	239 (97%)
Remelt, n (%)	3 (1%)
Retest n (%)	4 (2%)

## Discussion

We present here an assay development and validation study of a platform useful for rapid and simple SNP testing for patient stratification regimens. Exemplified herein, we identified *IL28B* genotypes using an IVD-certified assay that achieved accurate results as compared to the current standard TaqMan SNP genotyping tests. The PoC assay platform described provides clear advantages over conventional lab based testing: (i) it is non-invasive utilizing buccal swab samples instead of peripheral blood or plasma and thereby minimizing contamination risks; and (ii) it does not require trained specialists and can be implemented by a nurse or healthcare worker. The greatest advantage is the rapidity at which results are available—with total analysis time from sample collection to result in less than an hour—thus making it possible to inform same-day clinical decisions. This is particularly impactful in resource limited settings in low and low-medium income countries where patient time with healthcare professionals is restricted and access to certified laboratories can translate into delays of weeks—to—months [17]. The Genedrive PoC tests are therefore positioned to deliver modern molecular decentralized diagnostics to healthcare environments that currently lack access to standardized testing services.

The authors acknowledge that *IL28B* testing is no longer required in countries where DAA therapy is widely available [18]. Nonetheless, the strong predictive power of *IL28B* SNPs on spontaneous clearance and response to IFN-based therapies may still be relevant in resource limited settings[8],[19] including countries such as China with a high burden of HCV and predominantly (60%) CC *IL28B* genotypes[20]. In countries where healthcare budgets do not currently support these treatments it remains an open question as to whether clinicians will continue to use interferon-based treatments where *IL28B* testing could play an important role. Additionally, *IL28B* polymorphisms have been reported to be predictive for transition to cirrhosis [21], fibrosis [22], hepatocellular carcinoma [23],[24], and potentially to influence clinical responses in HBV infection [25], HAV infection [26] and glucose metabolism in type 2 diabetes [27]. Furthermore due to the distinct global distribution of *IL28B* alleles, and the fact that *IL28B* has the highest reported diversity of all interferon subtypes having undergone positive selection in European and African populations [28], it seems likely that *IL28B* polymorphisms are important determinants in other pathological situations. One recent study that supports this concept reported an innate antiviral gene signature in monocytes after childbirth that could be stratified according to *IL28B* polymorphisms [29].

In sum, we report the development of the first CE-IVD certified point of care SNP test, establishing a general path towards decentralized SNP genotype-based precision medicine. This represents a first step towards delivering state-of-the-art diagnostic tools to positively impact global health.
